# The Impact of Obesity on Diabetes Onset and Neovascularization in Mouse Models of Metabolic Stress

**DOI:** 10.3390/ijms25021214

**Published:** 2024-01-19

**Authors:** Sai Pranathi Meda Venkata, Hainan Li, Liping Xu, Jie-Mei Wang

**Affiliations:** 1 Department of Pharmaceutical Sciences, Eugene Applebaum College of Pharmacy and Health Sciences, 259 Mack Ave, 3122 Applebaum Building, Detroit, MI 48201, USA; saipranathi@wayne.edu (S.P.M.V.); lxu@wayne.edu (L.X.); 2 Centers for Molecular Medicine and Genetics, Wayne State University, Detroit, MI 48201, USA; 3 Karmanos Cancer Institute, Detroit, MI 48201, USA

**Keywords:** hyperglycemia, dyslipidemia, animal model, neovascularization, sexual dimorphism

## Abstract

Animal models of metabolic disorders are essential to studying pathogenic mechanisms and developing therapies for diabetes, but the induction protocols vary, and sexual dimorphism often exists. In a chronic diabetic model of diet-induced obesity (DIO) and low-dose streptozotocin (STZ)-induced hyperglycemia, blood glucose and lipid profiles were measured. The high-fat (HF) diet damaged insulin sensitivity and increased triglycerides, total cholesterol, LDL-cholesterol, HDL-cholesterol, and liver lipid deposition. STZ increased blood glucose and liver fibrosis with less effects on blood lipids or liver lipid deposition. The combination of DIO and STZ treatments led to significant liver lipid deposition and fibrosis. Female mice showed delayed body weight gain on HF diet and resisted STZ-induced hyperglycemia. However, once they developed DIO, which occurs around 26 weeks of HF diet, the female mice were prone to STZ-induced hyperglycemia. In hindlimb ischemia, male mice in the DIO-STZ group showed significantly worse neovascularization compared with DIO or STZ groups. The DIO-STZ females showed significantly worse recovery than the DIO-STZ males. Our observations suggest that DIO-STZ is a plausible model for studying metabolic and cardiovascular disorders in obesity and diabetes. Moreover, the findings in female animals stress the need to assess sexual dimorphism and investigate the underlying mechanisms that contribute to the worse vasculopathy manifestations in females in metabolic models.

## 1. Introduction

Diabetes mellitus is a multifactorial disease that affects almost all organs and systems in our body. The research for understanding the pathogenic mechanisms and the development of therapeutic approaches for diabetes and diabetes-related complications have heavily relied on animal models. Over the decades, multiple animal models have been developed to induce obesity and related metabolic disorders in rodents, such as spontaneous genetic mutations, high-energy diets, and chemical ablation of the pancreatic beta cells [1].

While no single model replicates all aspects of diabetes, animals fed on a high-fat (HF) diet demonstrate many pathologies of metabolic syndromes, such as obesity (especially abdominal obesity), mild hyperglycemia, insulin resistance, exhausted islet function, hormonal disorders, adipose depots, nephropathy, retinopathy, and neuropathy [2]. Several contributing factors, such as sex, age, type of diet, and health status, can significantly affect the animals’ responsiveness to HF diet [3]. A compensatory method to induce a more confirmative phenotype of hyperglycemia is to administrate streptozotocin (STZ), an antibiotic particularly toxic to islet beta cells [4]. Therefore, the combination of HF diet-induced obesity (DIO) and low-dose STZ administrations has been developed to replicate type 2 diabetes (T2D) [5]. Despite the wide variety in the HF and the STZ treatment protocols, the combination of DIO and STZ procedures is a reasonable T2D animal model representing a relatively late stage of diabetes with decompensated β-cell function [6].

Vascular disorders are the most prominent comorbidities and commonalities of diabetes [7]. Ideally, an animal model of diabetic vascular disease should exhibit accelerated atherosclerosis, coronary arterial diseases, retinopathy, nephropathy, and peripheral vascular disease (PAD), manifested as aberrant neovascularization capacities in the tissues. To date, limited information is available for researchers to assess the severity of vascular disorders in HF and STZ models because these models were often combined with various regimens or conducted in genetically modified animals. Furthermore, female animals were often left out of these models because they resist diet-induced lipid disorders, weight gain, or STZ-induced hyperglycemia, and female animals have significantly delayed cardiovascular manifestations.

This study compares the blood glucose, lipid panels, and liver pathologies among animal models of STZ, DIO, and DIO-STZ using C57BL/6 mice. Hindlimb ischemia was used to evaluate their revascularization capacity. Importantly, we prolonged the duration of the protocol to include female animals that met the criteria for T2D in the study and tested their neovascularization ability. While each animal model of diabetes has its strengths and limitations, our data suggest that HF diet-induced obesity combined with STZ-induced hyperglycemia best represents the metabolic and cardiovascular disorders found in the human population in the worldwide prevalence of obesity and diabetes. We believe that the observations from this study will provide referential information for researchers in metabolic and cardiovascular studies.

## 2. Results

### 2.1. The Body Weight Gain, Hyperglycemia, and Insulin Resistance in Metabolic Models in Male Mice

**Figure 1:**
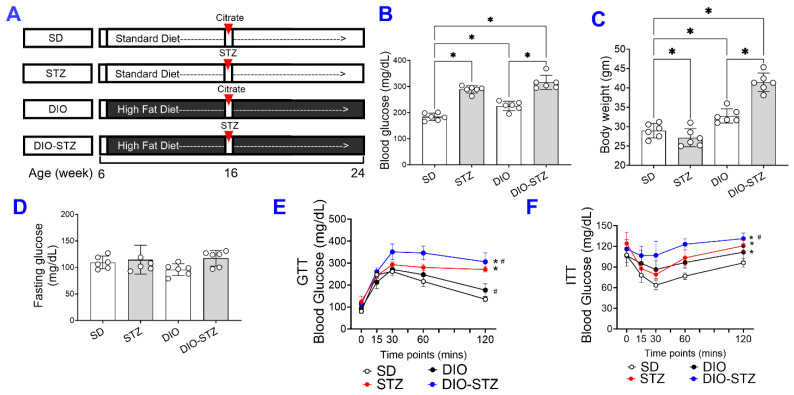
The body weight gain, hyperglycemia, and insulin resistance in male mice with either standard diet (SD), high-fat diet-induced obesity (DIO), low-dose streptozotocin (STZ)-induced hyperglycemia, or type 2 diabetes (T2D) induced by both DIO plus low-dose STZ. (**A**) Protocols used to develop animal models of chronic conditions, including hyperglycemia induced by low-dose STZ injections, DIO, and T2D induced by both DIO plus low-dose STZ injections using SD plus vehicle (citrate buffer) injection as healthy control. Blood glucose levels (**B**) and Body weight (**C**) of male mice on SD, low dose STZ, DIO, and T2D by DIO-STZ groups. *n* = 6 per group. * *p* < 0.05; (**D**) The fasting blood glucose levels of male mice on SD, low dose STZ, DIO, and T2D induced by DIO-STZ groups. *n* = 6 per group. ns, not significant. (**E**) Blood glucose recordings of male mice after a glucose tolerance test (GTT). *n* = 6 in each group. Each animal was given an intraperitoneal injection of glucose at 2.5 g/kg body weight. * *p* < 0.05. vs. SD; ^#^ *p* < 0.05 vs. STZ. (**F**) Blood glucose recordings of male mice after an insulin tolerance test (ITT). *n* = 6 per group. * *p* < 0.05 vs. SD; ^#^ *p* < 0.05 vs. STZ. Each animal was given an intraperitoneal insulin injection at 0.75 U/kg body weight, followed by blood glucose monitoring. Data are represented as mean ± SD. When a comparison was made between the two groups, the *p* values were determined using the Mann–Whitney U test. When comparison was made between more than two groups of treatments, the *p* values were first determined by the Kruskal–Wallis test across all the groups and, if significant, the pairs of primary interest, based on scientific rationale, were assessed using the Mann–Whitney U test with Benjamini Krieger, and Yekutieli’s adjustment for multiple comparisons. The significant differences that came from post-hoc comparisons of groups were noted.

We created an obese and chronic hyperglycemia (DIO-STZ) model in male and female mice using the schema highlighted in Figure 1A. In this model, DIO was induced for over 10 weeks before animals were rendered hyperglycemic using low-dose STZ, and the animals were kept for an additional 8 weeks (~2 months) on a continued HF diet. A standard diet (SD) with STZ injections served as a hyperglycemic group, and DIO with citrate buffer injections served as a nondiabetic obese control. The male animals following such protocol developed desired phenotypes of T2D. The body weight and blood glucose of all the groups are shown in Table 1. As shown in Figure 1B, the basal blood glucose levels of male DIO mice were higher than those of the male-SD mice. The blood glucose levels were significantly increased after the STZ injections with or without HF diet. On the other hand, the body weight was increased by the HF diet, as shown in the DIO or DIO-STZ group (Figure 1C). However, STZ administration reduced the body weight in males fed with standard chew (shown as STZ group) while increasing body weight in males with HF diet (DIO-STZ). Although we did not observe a significant difference in the fasting blood glucose levels of male mice under SD, STZ, DIO, and DIO-STZ treatments (Figure 1D), there was a significantly impaired glucose tolerance (Figure 1E) and insulin tolerance (Figure 1F) in the male-DIO-STZ followed by male + DIO.

### 2.2. Lipid Panels and Hepatic Steatosis in Metabolic Models in Male Mice

**Figure 2:**
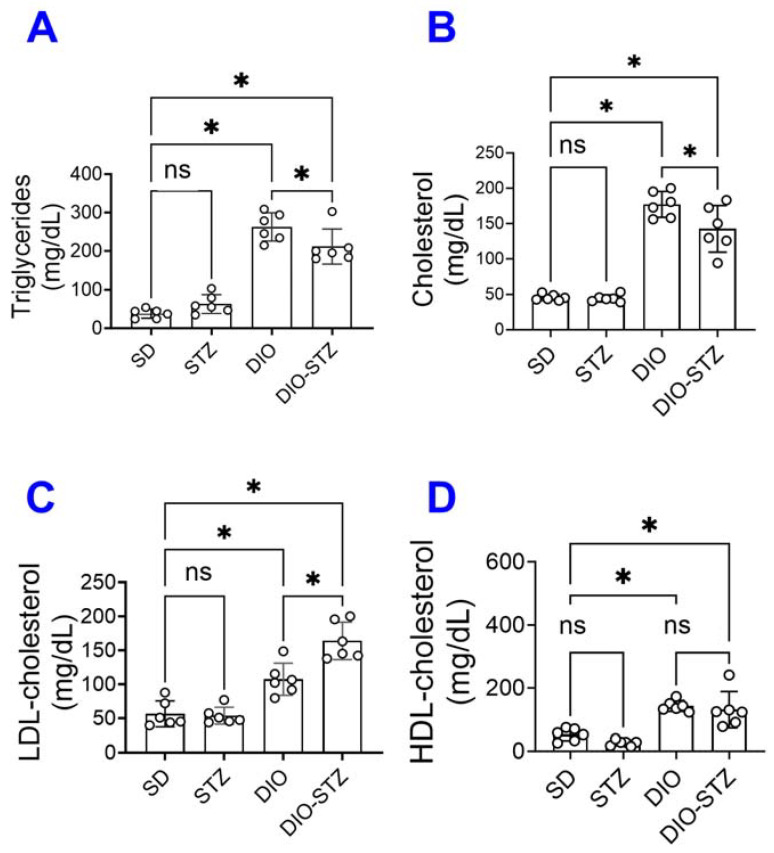
Blood lipid panel of mice with either standard diet (SD), high-fat diet-induced obesity (DIO), low-dose streptozotocin (STZ)-induced hyperglycemia, or type 2 diabetes (T2D) induced by both DIO plus low-dose STZ. (**A**) Triglyceride levels, (**B**) Total cholesterol, (**C**) Low-density lipoprotein levels (LDL-cholesterol), and (**D**) High-density lipoprotein levels (HDL-cholesterol) were measured in SD, STZ, DIO, DIO-STZ treatment groups. *n* = 6 per group. * *p* < 0.05, ns, not significant. In all the figures, data are represented as mean ± SD. The *p* values were first determined by the Kruskal–Wallis test across all the groups. If significant, the pairs of primary interest, based on scientific rationale, were assessed using the Mann–Whitney U test with Benjamini, Krieger, and Yekutieli’s adjustment for multiple comparisons. The significant differences that came from post hoc comparisons of groups were noted.

**Figure 3:**
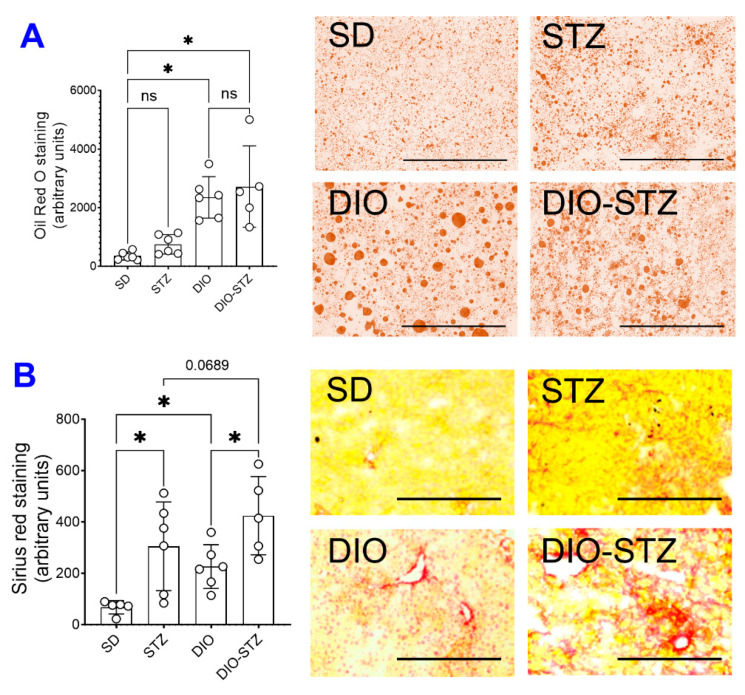
Liver lipid deposition and fibrotic development of mice with either standard diet (SD), high-fat diet-induced obesity (DIO), low-dose streptozotocin (STZ)-induced hyperglycemia, or type 2 diabetes (T2D) induced by both DIO plus low-dose STZ. (**A**) Liver tissues of the mice from SD, STZ, DIO, and DIO-STZ treatment groups were fixed and stained with Oil red O staining to visualize the lipid deposition. Oil Red O staining showed the presence of lipid droplets (colored red) of various sizes. *n* = 6 per group, * *p* < 0.05, ns, not significant. Scale bar, 400 μm. HPF, high-power field. (**B**) Liver tissues from SD, STZ, DIO, and DIO-STZ treatment groups were fixed and performed with the Picro Sirius staining to analyze the tissue for fibrosis. *n* = 6 per group, * *p* < 0.05. Scale bar, 200 μm. HPF, high-power field. Representative images are shown in the right panel. In all the figures, data are represented as mean ± SD. The *p* values were first determined by the Kruskal–Wallis test across all the groups. If significant, the pairs of primary interest, based on scientific rationale, were assessed using the Mann–Whitney U test with Benjamini, Krieger, and Yekutieli’s adjustment for multiple comparisons. The significant differences that came from post hoc comparisons of groups were noted.

Since an HF diet significantly affects lipid metabolism, we measured the lipid panels in the blood. As shown in Figure 2, the levels of triglycerides (Figure 2A), total cholesterol (Figure 2B), LDL-cholesterol (Figure 2C), and HDL-cholesterol (Figure 2D) were significantly increased by the HF diet, regardless of STZ administrations. On the other hand, STZ administration did not cause changes in blood lipid panels in male animals on a standard diet, but in male animals on an HF diet, it significantly increased LDL-cholesterol while decreasing triglycerides and cholesterol. Regarding hepatic steatosis, the HF diet induced a significant amount of lipid deposition (Figure 3A) and enhanced liver fibrosis (Figure 3B). The STZ administration did not further increase lipid deposition in the liver, regardless of diet groups (Figure 3A), but significantly increased fibrosis (Figure 3B). These data suggested that the male mice developed chronic hyperlipidemia, fatter liver, and liver fibrosis when put on an HF diet. While STZ could aggregate the hyperlipidemia and liver fibrosis in these animals, the liver lipid deposition was unaffected.

### 2.3. Female Mice Are Resistant to Metabolic Models

**Figure 4:**
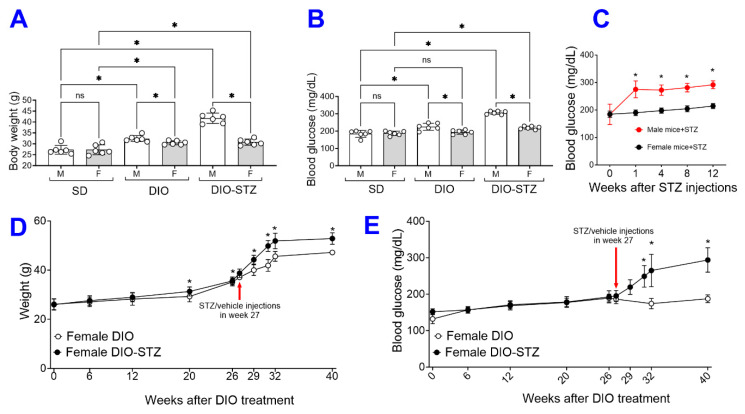
The female mice with either standard diet (SD), high-fat diet-induced obesity (DIO), low-dose streptozotocin (STZ)-induced hyperglycemia, or type 2 diabetes (T2D) induced by both DIO plus low-dose STZ. Comparison of the (**A**) body weights and (**B**) blood glucose levels between male and female mice on SD, DIO, and T2D induced by DIO-STZ groups. (**C**) Blood glucose levels of mice of both sexes at various time points after STZ injections. *n* = 6 per group. * *p* < 0.05 vs. females receiving the same procedure at the same time point. Body weights (**D**), Blood glucose levels (**E**) of female DIO mice receiving procedures of DIO and PBS, DIO and STZ-induced hyperglycemia over time. *n* = 10 to 16 per group. When a comparison was made between the two groups, the *p* values were determined by the Mann–Whitney U test. When comparison was made between more than two groups of treatments, the *p* values were first determined by the Kruskal–Wallis test across all the groups and, if significant, the pairs of primary interest, based on scientific rationale, were assessed using the Mann–Whitney U test with Benjamini Krieger, and Yekutieli’s adjustment for multiple comparisons. The significant differences that came from post hoc comparisons of groups were noted.

Following the protocol in Figure 1A, we attempted to create these models of metabolic stress in female animals. As shown in Figure 4A, the average body weight of the female mice on the HF diet was moderately increased for 16 weeks, but it was much more drastic in male mice. The blood glucose of female mice was not raised by the HF diet and was only moderately increased by the HF diet combined with STZ treatment (Figure 4B). At each checkpoint (DIO at 16 weeks and DIO + STZ at 24 weeks), there were significant differences in body weight and blood glucose between males and females. In addition, the female mice remained normoglycemia upon receiving STZ injections. At the same time, their male counterparts developed hyperglycemia within the first week post-injections and stayed hyperglycemia after that (Figure 4C). This sexual dimorphism in response to an HF diet or STZ-induced hyperglycemia was consistent with previous reports (6). Estrogen has been speculated to play a protective role in metabolic homeostasis against nutrient stress [8]. We then extend the HF diet to these female mice. Our results showed that most female mice started gaining body weight around 26 weeks into the HF diet. The animals were given the low-dose STZ injections or citrate buffer in week 27. The female mice developed obesity and hyperglycemia over the next eight weeks compared with vehicle-injected animals (female-DIO mice) and were stabilized at week 40 (Figure 4D,E).

**Figure 5:**
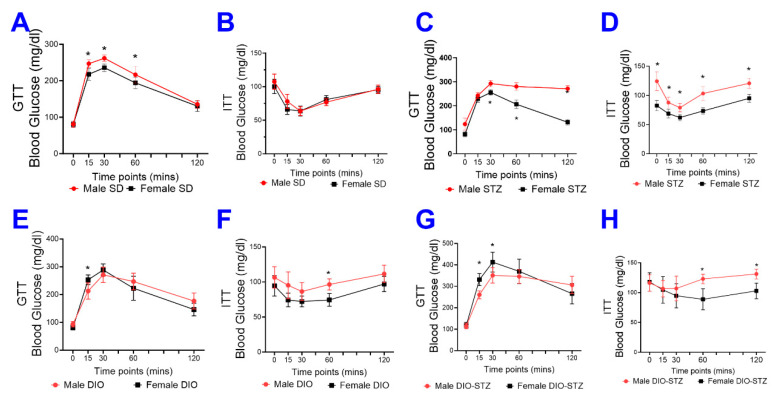
Blood glucose recordings of glucose tolerance test (GTT) and insulin tolerance test (ITT) in male and female mice with either standard diet (SD), high-fat diet-induced obesity (DIO), low-dose streptozotocin (STZ)-induced hyperglycemia, or type 2 diabetes (T2D) induced by both DIO plus low-dose STZ injections. Comparison of blood glucose recordings of male and female mice in GTT in SD (**A**), STZ (**C**), DIO (**E**), and DIO-STZ (**G**) groups. *n* = 6 per group, * *p* < 0.05. Each animal was given an intraperitoneal injection of glucose at 2.5 g/kg body weight. Comparison of blood glucose recordings of male and female mice in ITT in SD (**B**), STZ (**D**), DIO (**F**), and DIO-STZ (**H**) groups. *n* = 6 per group, * *p* < 0.05. Each animal was given an intraperitoneal insulin injection at 0.75 U/kg body weight, followed by blood glucose monitoring. When a comparison was made between the two groups, the *p* values were determined by the Mann–Whitney U test. When comparison was made between more than two groups of treatments, the *p* values were first determined by the Kruskal–Wallis test across all the groups and, if significant, the pairs of primary interest, based on scientific rationale, were assessed using the Mann–Whitney U test with Benjamini Krieger, and Yekutieli’s adjustment for multiple comparisons. The significant differences that came from post hoc comparisons of groups were noted.

We also compared the glucose tolerance test (GTT) and insulin tolerance test (ITT) in female and male animals of SD, DIO, STZ, and DIO-STZ models. In SD groups, females demonstrated superior glucose tolerance than males (Figure 5A), but the insulin tolerance was comparable between females and males (Figure 5B). In the STZ model, females demonstrated superior glucose tolerance and insulin sensitivity compared with males (Figure 5C,D). In the DIO model, the glucose tolerance was comparable between females and males (Figure 5E), and females demonstrated slightly superior insulin sensitivity compared with males (Figure 5F). Similarly, in the DIO-STZ model, female and male mice demonstrated comparable glucose tolerance (Figure 5G), but females demonstrated superior insulin sensitivity compared with males (Figure 5H).

### 2.4. Neovascularization in Mouse Models of Metabolic Stress in Both Male and Female Mice

**Figure 6:**
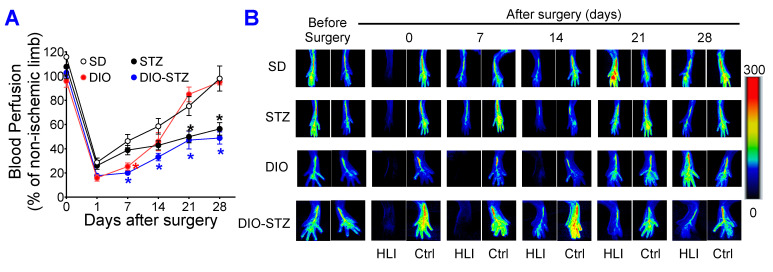
Blood perfusion recovery of mice with either standard diet (SD), high-fat diet-induced obesity (DIO), low-dose streptozotocin (STZ)-induced hyperglycemia, or type 2 diabetes (T2D) induced by both DIO plus low-dose STZ injections after hindlimb ischemia. After the animal models of chronic conditions, including hyperglycemia induced by low-dose STZ injections, DIO, and T2D induced by DIO-STZ using SD plus vehicle (citrate buffer) injection as healthy control, was developed, hind limb ischemia was created in these animals using unilateral artery ligation. A laser Doppler Imager was used to monitor the blood perfusion during the recovery phase (4 weeks). The perfusion index was calculated by the percentage of blood perfusion read in the ischemic limb compared with that in the sham surgery-operated limb in the same animal. (**A**) Blood perfusion in the mice with STZ-induced hyperglycemia, with or without DIO. *n* = 6 per group. * *p* < 0.05 vs. SD at the same time point. The color of the (*) corresponded to the group that SD was compared with. Representative images of blood perfusion are shown in (**B**). HLI, hind limb with ischemia, Ctrl, control limb. Data are presented as mean ± SD. When a comparison was made between the two groups, the *p* values were determined by the Mann–Whitney U test. When comparison was made between more than two groups of treatments, the *p* values were first determined by the Kruskal–Wallis test across all the groups and, if significant, the pairs of primary interest, based on scientific rationale, were assessed using the Mann–Whitney U test with Benjamini Krieger, and Yekutieli’s adjustment for multiple comparisons. The significant differences that came from post hoc comparisons of groups were noted.

**Figure 7:**
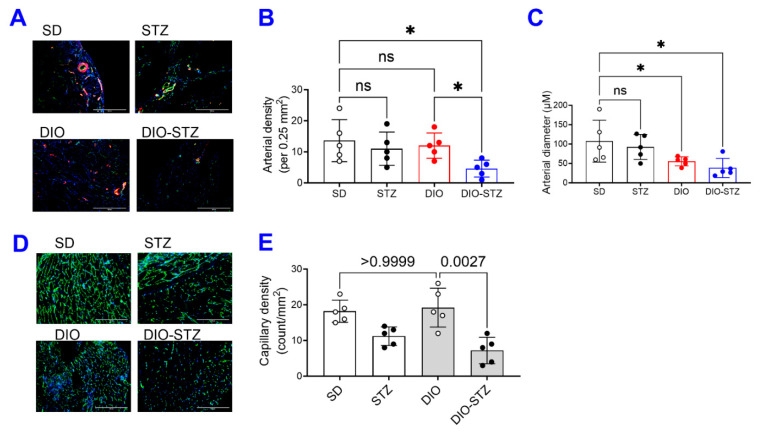
Collateral formation of mice with either standard diet (SD), high-fat diet-induced obesity (DIO), low-dose streptozotocin (STZ)-induced hyperglycemia, or type 2 diabetes (T2D) induced by both DIO plus low-dose STZ injections after hindlimb ischemia. After the animal models of chronic conditions, including hyperglycemia induced by low-dose STZ injections, DIO, and T2D induced by DIO-STZ using SD plus vehicle (citrate buffer) injection as healthy control, was developed, hind limb ischemia was created in these animals by unilateral artery ligation. (**A**) The adductor muscle was collected at the end of the study and performed α-Smooth muscle actin (αSMA, red) and CD31 staining (green). Scale bar, 400 μm. The quantification of arterial density (**B**) and diameter (**C**) are shown. * *p* < 0.05. ns, not significant. (**D**) The calf muscle was collected and stained with CD31 antibody. Scale bar, 400 μm. The quantification of capillary density is shown in (**E**). When a comparison was made between the two groups, the *p* values were determined by the Mann–Whitney U test. When comparison was made between more than two groups of treatments, the *p* values were first determined by the Kruskal–Wallis test across all the groups and, if significant, the pairs of primary interest, based on scientific rationale, were assessed using the Mann–Whitney U test with Benjamini Krieger, and Yekutieli’s adjustment for multiple comparisons. The significant differences that came from post hoc comparisons of groups were noted.

**Figure 8:**
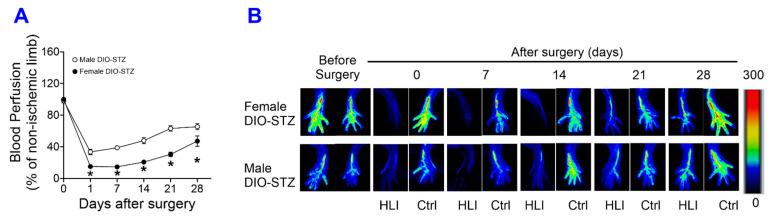
Blood perfusion recovery of male and female mice with either standard diet (SD), high-fat diet-induced obesity (DIO), low-dose streptozotocin (STZ)-induced hyperglycemia, or type 2 diabetes (T2D) induced by both DIO plus low-dose STZ injections after hindlimb ischemia. After the animal models of chronic conditions, including hyperglycemia induced by low-dose STZ injections, DIO, and T2D induced by DIO-STZ using SD plus vehicle (citrate buffer) injection as healthy control, was developed, hind limb ischemia was created in these animals by unilateral artery ligation. A laser Doppler Imager was used to monitor the blood perfusion during the recovery phase (4 weeks). The perfusion index was calculated by the percentage of blood perfusion read in the ischemic limb compared with that in the sham surgery-operated limb in the same animal. (**A**) Blood perfusion recovery in male and female DIO-STZ groups after hind limb ischemia. *n* = 6 per group. * *p* < 0.05. Representative images of blood perfusion are shown in (**B**). HLI, hind limb with ischemia, Ctrl, control limb. Data are presented as mean ± SD. When a comparison was made between the two groups, the *p* values were determined by the Mann–Whitney U test. When comparison was made between more than two groups of treatments, the *p* values were first determined by the Kruskal–Wallis test across all the groups and, if significant, the pairs of primary interest, based on scientific rationale, were assessed using the Mann–Whitney U test with Benjamini Krieger, and Yekutieli’s adjustment for multiple comparisons. The significant differences that came from post hoc comparisons of groups were noted.

The recovery of blood flow in ischemic limbs measured by laser Doppler is a gold standard approach to evaluate the capability of neovascularization in vivo. As shown in Figure 6A,B, in male mice, the blood flow was fully recovered in the SD and DIO groups at the end of the experiment. The DIO group showed a significantly delayed increase in blood flow in the early days but quickly caught up with the SD group. In contrast, the STZ and DIO-STZ groups showed impaired blood perfusion throughout the recovery period. STZ group showed a comparable pace in blood flow in the early days but did not reach full recovery. The DIO-STZ group showed the worst blood flow recovery among all four groups. At the end of the study, the adductor muscle was collected for collateral formation in the thigh using α-Smooth muscle actin (αSMA, red) and CD31 staining (green) (Figure 7A). Our data suggested that the DIO-STZ group had reduced arterial density (Figure 7B) and diameter (Figure 7C) compared with the SD group. In contrast, the DIO group had reduced arterial diameter, and the STZ group displayed sufficient collaterals compared with the SD group. In the meantime, capillary formation was evaluated in the calf muscle using CD31 staining (green, Figure 7D). Our data suggested a moderate capillary density reduction in the STZ group compared with the SD group (Figure 7E). The DIO-STZ group had the least capillary density. We also evaluated the recovery of blood perfusion in the ischemic limb in female mice that reached the criteria of DIO-STZ phenotypes. As shown in Figure 8A,B, these DIO-STZ female mice demonstrated worse blood perfusion than the male DIO-STZ males throughout the recovery period.

## 3. Discussion

This study compared glucose metabolic profiles among metabolic stress models, including DIO, STZ, and T2D in male and female mice. Lipid profiles have been reached among metabolic stress models in male mice. Our data suggested that the DIO model produces insulin resistance, mild hyperglycemia, and liver steatosis as major risk factors for diabetes onset. With STZ-induced insulin deficiency exacerbating hyperglycemia, the DIO-STZ model can recapture the diabetic phenotypes, effectively mimicking the progression of diabetes and its associated vascular relatively quickly. A prolonged HF diet can compromise the resistance to STZ-induced hyperglycemia in female mice. When rendered T2D, these females demonstrated worse ischemia-induced neovascularization than males.

Hepatic lipid accumulation (steatosis) is a prominent feature of nonalcoholic fatty liver disease (NAFLD), characterized by the accumulation of lipids, mainly triglycerides, in the cytoplasm of hepatocytes. Liver fibrosis, characterized by excessive accumulation of extracellular matrix (predominantly collagen), often occurs in advanced fatty liver due to lipotoxicity, inflammation, and cell stress [9]. Recently, it has been proposed that the term NAFLD be revised to metabolic dysfunction-associated fatty liver disease (MAFLD), a multisystem disease characterized by the presence of hepatic steatosis, T2D, and obesity [10]. Increasing clinical evidence suggests that NAFLD/MAFLD are associated with extrahepatic manifestations, mostly cardiovascular and cancer events [11]. The rationale behind this is that MAFLD better reflects the pathogenetic basis of the disease and better explains the mutual interplay between fatty liver and metabolic changes [12], emphasizing fatty liver as an independent risk factor via promoting a systemic pro-atherosclerotic, proinflammatory, and profibrotic environment [13,14]. In this regard, animal models of MAFLD are valuable to investigate the causal relationship between fatty liver, atherosclerosis, and cardiovascular dysfunction.

A critical finding from this study is the decreased sexual dimorphism in response to STZ-induced hyperglycemia in animals with established DIO. It has been known that female mice, often reported in the most commonly used C57BL/6 mouse lines, are resistant to low-dose STZ-induced hyperglycemia [15,16] and HF diet-induced metabolic syndrome [17,18], while their male counterparts are vulnerable. This sexual dimorphism has led to the exclusion of female mice in many metabolic studies. An alternative strategy to include female mice is to increase the STZ dosage or repeat the low-dose injections [19]. Our data have shown that when put on an HF diet for more than 6.5 months (26 weeks), the female mice started to accelerate body weight gain and increase blood glucose. They are prone to STZ-induced hyperglycemia, similar to the male mice, suggesting that preconditioning of obesity has eliminated the sexual dimorphism and that this protocol could be feasible to establish a T2D model in adult female mice. Most importantly, female DIO-STZ mice demonstrated worse neovascularization than male DIO-STZ mice. Please note that there was an age difference between male DIO-STZ mice (24 weeks) and female DIO-STZ mice (40 weeks), which could potentially contribute to inferior neovascularization in females. A better control male group would be a prolonged HF diet (26 weeks) before STZ administration, as we recognize this is one of the study’s limitations. Nonetheless, our data suggests that obesity might fundamentally alter the regulation of metabolic homeostasis in females, making them prone to hyperglycemia and PAD. This is clinically relevant because PAD often remains underdiagnosed and undertreated when the patients are asymptomatic, and women are particularly vulnerable because women receive delayed or even deferred clinical recognition compared with men despite a similar or higher prevalence of PAD [20]. Further studies to elucidate the underlying mechanisms driving these metabolic and cardiovascular differences between males and females will shed light on the development of preventative and therapeutic approaches in the population at risk.

It was interesting to note that the size and variation of lipid droplets (LDs) in the DIO group were more remarkable than that in the DIO-STZ group based on oil red O staining (Figure 3); under the HF diet, the significant LD often indicates hepatocytes’ capacity to cope with lipotoxicity because large LDs are relatively inactive compared with small ones [21,22]. The action of STZ on liver LD was not entirely consistent with its action on blood lipid panels. In our study, STZ on the normal diet, which was considered to mimic the insulin deficiency in type 1 diabetes (T1D), induced marginal changes in lipid profiles. This was consistent with findings in T1D patients in the clinic [23] but not with previous animal studies using the STZ model that observed an increase in triglycerides [24,25]. However, STZ added to the HF diet caused a decrease in triglycerides and cholesterol and an increase in LDL-cholesterol, while HDL-cholesterol was not altered (Figure 2). In the meantime, DIO-STZ mice seemed to have altered LD catabolism, leading to small and diffusive LD distribution in the liver. The variation in STZ protocols, with or without atherogenic diet or genetic background of the animals, might contribute to the discrepancies found in the blood lipid profiles among reports, which warrant future investigations. In a recent report, Willecke F, et al. propose that STZ modulates lipolysis instead of hepatic lipogenesis, in addition to its recognized pharmacological action of destroying pancreatic β cells and, therefore, decreasing insulin secretion [25]. This study sheds light on a potential mechanism for the altered lipid profiles in the STZ-induced hyperglycemic model. On the other hand, LD catabolism is mediated by highly dynamic and active lipolysis and lipophagy involving multiple pathways and machinery [26,27]. Mechanistic investigations into the molecular pathways in these two processes will provide informative data for researchers who will be or are currently using this DIO-STZ model. In addition, we did not have data on adipose tissue deposition. In future studies, obtaining the fat mass and systemic adipose tissue deposition among these metabolic models in both female and male animals would be necessary.

Hind limb ischemia is the most commonly used preclinical model for PAD and critical limb ischemia, but many variations in the model, including the method of occlusion, the number of occlusions, and the position at which the occlusions are made to induce ischemia [28]. Our surgical procedure used the scission of the femoral and saphenous arteries. This model produces more severe ischemia that can cause both collateral opening (arteriogenesis) in the thigh as well as capillary formation (angiogenesis) in the calf, according to previous reports [29], compared with the femoral artery ligation model that produces moderate ischemia which induces arteriogenesis predominantly in the thigh, with minimal calf angiogenesis. Our data suggested that in male mice, the DIO-STZ group had the worst collaterals and capillaries among all four groups, mimicking the situation in the clinic. The STZ group had a faster blood flow recovery within one week but remained in impaired perfusion after that. On the other hand, DIO mice had low blood perfusion in the first week but were able to catch up during the last two weeks for better blood perfusion than the STZ groups. Our histological data can help to explain these unparalleled healing curves. Although the tissue was collected at the end of the experiment, in STZ mice, the collateral formation in the thigh was significant, but the capillary formation in the calf was low (Figure 7A–C). In DIO mice, the collaterals in the thigh were small, but capillary formation in the calf was robust. Considering that collateral formation occurs sooner than capillary formation upon ischemia injury, the histological data was consistent with the blood perfusion detected by laser Doppler. Nonetheless, verification of these changes at the early time point after injury, for example, on day 7, is needed in future studies.

In this study, we used C57BL/6 mice, the most popular strain for biomedical studies on athetosis, birth defects, metabolism, and cancer, due to their low cost, low incidence of spontaneous tumorigenesis, and prone to develop obesity, diabetes, and atherosclerosis induced by diet [30]. However, mouse strain might affect the onset or pathological deposition of the diabetes model. For example, FVB/N mice become more glucose intolerant but present lower epididymal fat accumulation when fed a high-fat diet [31,32]. One suggestion is to create these metabolic models in diverse outbred animals that could demonstrate the heterogeneity of body weight gain, hyperinsulinemia, hyperglycemia, and hyperleptinemia, which are recapitulated in humans [33].

## 4. Materials and Methods

### 4.1. Animals

The C57BL/6 male and the female mice were purchased from Jackson Laboratory. The animals were housed in the animal facility under standardized conditions and followed a 12 h/12 h light/dark cycle. All animal experiments were performed according to Wayne State University Institutional Animal Care and Use Committee (IACUC) guidelines (IACUC number: 23-05-5829, approved date: 10 August 2023).

### 4.2. Streptozotocin (STZ)-Induced Chronic Hyperglycemic Model

To create an animal model of chronic hyperglycemia, we injected the animals with STZ at 50 mg/kg for five consecutive days (STZ group). The control groups were injected with citrate buffer (pH = 4.5). After confirming that the animals were hyperglycemic (≥250 mg/dL), they were maintained for at least 2 months.

### 4.3. Diet-Induced Obesity (DIO) Model

The animals were housed in the animal facility under standardized conditions and had free access to water. The animals receiving DIO procedures will be on an HF diet (60% kcal% fat, D12492; Research diets). The control group received a standard mouse diet (11.5% kcal% fat, LabDiet, Brentwood, MO, USA, 5L0D), as a previous report has shown no significant differences in phenotypic, metabolic outcomes between the standard diet and the purified low-fat diet in C57BL/6 mice [34]. The animals remained on the diet for 10 weeks before they received any add-on treatment.

### 4.4. DIO-STZ-Induced T2D Model

The animals were maintained on a high-fat diet (60% fat Research Diets, D12492) for 10 weeks. Then, they received additional low-dose STZ via intraperitoneal injections for 7 days (day 1 at 50 mg/kg, days 2–7 at 25 mg/kg). After confirming that these animals were hyperglycemic (≥250 mg/dL), they were maintained for two months before use in experiments [35]. The HF diet + STZ model is a simple way to generate an obese T2D model with moderate hyperglycemia and insulin deficiency. The overall treatment protocols are demonstrated in Figure 1A.

### 4.5. Glucose Tolerance Test

Mice were fasted for 4 h (morning fast). Following the fast, sufficient volume of 250 mg/mL, glucose solution was administered intraperitoneally at 2.5 g/Kg body weight. The blood sample from each unrestrained mouse was taken by tail snipping for blood glucose measurements. Before the snips, the tail will be dipped into 0.25% bupivacaine for local anesthesia. A blood sample (5 μL or less) was collected before glucose administration (basal measurements) and then at 15, 30, 60, and 120 min after the glucose administration for the duration of the experiment.

### 4.6. Insulin Tolerance Test

Following 4 h of fast (morning fast), the animals were injected with 0.1 U/mL of insulin (Sigma-Aldrich, St. Louis, MO, USA, #10516) in PBS at 0.75 U/Kg body weights. The blood sample from each unrestrained mouse was taken by tail snipping for blood glucose measurements. Before the snips, the tail will be dipped into 0.25% bupivacaine for local anesthesia. A blood sample (5 μL or less) was collected before insulin administration (basal measurements) and then at 15, 30, 60, and 120 min after the insulin administration for the duration of the experiment.

### 4.7. Triglycerides Assay

Triglyceride Assays were performed using EnzyChrome^TM^ Triglyceride Assay Kit (BioAssay Systems, Hayward, CA, USA, ETGA-200) according to the manufacturer’s instructions. Briefly, plasma samples were diluted 5-fold in dH_2_O and then added with a Working Reagent containing Enzyme Mix, Lipase, ATP, and Dye reagent. The optical density at 570 nm was recorded after 30 min using a BioTek Epoch Spectrophotometer. A standard curve was created to convert the arbitrary units to the amount of triglycerides in serum using mg/dL as units.

### 4.8. Cholesterol Assay

Cholesterol Assay was performed using EnzyChrome^TM^ Cholesterol Assay Kit (BioAssay Systems, ECCH-100) according to the manufacturer’s instructions. The nicotinamide adenine dinucleotide (NAD) solution was added to standards and 10 times diluted samples plasma; the background optical density at 340 nm was recorded (OD0) using a BioTek Epoch Spectrophotometer. After adding the enzyme mix for 30 min, read the optical density again at 340 nm (OD30); the cholesterol concentrations were calculated from the standard curve by ΔOD (OD30-OD0).

### 4.9. High-Density Lipoprotein (HDL)-Cholesterol and Low-Density Lipoprotein (LDL)-Cholesterol Assay

HDL-Cholesterol and LDL-Cholesterol Assays were performed using Mouse HDL-Cholesterol (Crystal Chem, Elk Grove Village, IL, USA, 79990) and Mouse LDL-Cholesterol assay (Crystal Chem, 79980) according to the manufacturer’s instructions. Briefly, plasma samples and calibrator or control (0.9% saline) were mixed with polyvinyl sulfonic acid (PVS) and polyethylene-glycol methyl ether (PEGME) detergent (HDL-cholesterol assay) or PVS/PEGME enzyme solution (LDL-cholesterol assay) and equilibrated at 37 °C for 5 min. The absorbance at 600 nm was measured as OD_600 nm, A1/5 min_ using a BioTek Epoch Spectrophotometer. Then, the samples were added with Enzyme Solution (HDL-cholesterol assay) or Decomplexing Agent (LDL-cholesterol assay) and incubated at 37 °C for 5 min, and OD_600 nm, A2/10 min_ was measured at 600 nm. The HDL- or LDL-cholesterol concentration was calculated using the following equation: ΔA = OD_600 nm, A2/10 min_ − OD_600 nm, A1/5 min_; concentration = [(sample ΔA − blank ΔA)/(calibrator ΔA − blank ΔA)] × calibrator concentration.

### 4.10. Oil Red O Staining

Mouse liver samples were collected immediately after the animals were euthanized and embedded in OCT. An eight μm section was cut using a cryostat and directly transferred to poly-l-lysine coated slides (Fisher Scientific, Hampton, NH, USA, 12-550-15). Sections were fixed with 10% formalin at room temperature (RT) for 15 min and washed with running tap water for up to 10 min. The slides were rinsed with 60% isopropanol and then stained with freshly prepared Oil Red O stain (Sigma-Aldrich, St. Louis, MO, USA, O1391) working solution for 40 min. After washing 60% isopropanol and distilled water, the slides were mounted with an aqueous mounting reagent. The images were recorded by Inverted microscopy (EVOS, Thermo Fisher Scientific, Waltham, MA, USA).

### 4.11. Picro-Sirius Staining

Liver sections were fixed with 10% formalin at RT for 10 min and washed with water. After drying at room temperature for 10 min, the slides were hydrated with xylene and a graded series of alcohol solutions. Then, the slides were stained with Picro-Sirius Red solution (Abcam, Cambridge, UK, AB246832) for 30 min at room temperature. The excess staining was washed with 0.5% glacial acetic acid solution. The slides were dehydrated with 100% alcohol and xylene and mounted with a mounting medium (Electron Microscopy Science, Hatfield, PA, USA, 14800). The images were recorded by Inverted microscopy (EVOS, Thermo Fisher Scientific).

### 4.12. Hindlimb Ischemia Surgery

The hindlimb ischemia surgery was performed as in the previous study [35]. The femoral artery was isolated from the nerves and veins. The proximal portion of the femoral artery and the distal portion of the saphenous vein were ligated while the animal was under anesthesia. The arterial branches between the ligation were destroyed. The overlying skin was closed with sutures. Blood flow was measured before, immediately after, and 7, 14, 21, and 28 days after femoral arterial ligation using Laser Doppler imaging (PeriScan PIM 3 System, Perimed, Las Vegas, NV, USA). At the end of the study, muscle samples from hind limb ischemia studies were collected and embedded in OCT. An 8 μm section was cut using a cryostat and immediately transferred to poly-l-lysine coated slides (Fisher Scientific, 12-550-15). Immunofluorescent staining was performed as previously described [36]. The sections were incubated with a goat anti-mouse CD31 conjugated Alexa 488 antibody (R&D Systems, Minneapolis, MN, USA, #FAB 3628G) or incubated with a goat anti-mouse α smooth muscle actin (SMA) conjugated Alexa 594 antibody (Cell Signaling Technology, Danvers, MA, USA, #36110). Fluorescence images were recorded by fluorescence microscopy (EVOS, Thermo Fisher Scientific).

### 4.13. Statistical Analysis

All values are expressed as mean ± SD. For continuous variables that follow the normal distributions, such as blood glucose, body weight, and lipid panels, the statistical significance of differences between the two groups was determined by Student’s *t*-test. When more than two treatment groups were performed, one-way ANOVA was used across all the groups. If significant, pairs of focused groups were tested with two-sample *t*-tests, and *p* values were adjusted with Benjamini Krieger and Yekutieli’s method [37]. For continuous variables that failed Shiparo-Wilk normality tests, the statistical significance of differences between the two groups was determined using the Mann–Whitney U test. In blood perfusion curve data, the two groups were tested using multiple Mann-Whitney U tests with Benjamini Krieger and Yekutieli’s adjustment [37]. When more than two groups of treatments were performed, the Kruskal–Wallis test was applied across all the groups, and if significant, the pairs of primary interest, based on scientific rationale, were assessed using the Mann–Whitney U test with Benjamini Krieger and Yekutieli’s adjustment for multiple comparisons [37]. The significant differences that came from post-hoc comparisons of groups were noted. The post-hoc gatekeeping approaches and the adjustments preserved alpha spending and controlled false positive rate inflation due to multiple hypothesis testing. A value of *p* < 0.05 was considered statistically significant. The statistical analyses were performed using GraphPad Prism 9 (GraphPad Software 9.0).

## 5. Conclusions

In conclusion, our animal studies have demonstrated the essentiality of diet-induced obesity to the development of T2D and T2D-related impaired neovascularization. More importantly, our studies suggested that female mice have a delayed start of high-fat diet-induced obesity. Once they develop obesity, the female mice are prone to STZ-induced hyperglycemia and have worse neovascularization than the male mice. We believe this study provides valuable information for researchers to select the appropriate animal models that will continue to play a critical role in basic and translational research. Uncovering the diminished sex dimorphism in animals with obesity and the vulnerability of female obese animals to PAD may have important implications for developing protocols for preventing and managing cardiovascular complications in humans with metabolic stress.

## Figures and Tables

**Table 1:** The body weight, blood glucose, and blood lipid panels in animals with a standard diet (SD), high-fat diet-induced obesity (DIO), low-dose streptozotocin (STZ)-induced hyperglycemia, or type 2 diabetes induced by both DIO plus low-dose STZ injections (DIO-STZ).

Model	SD	STZ	DIO	DIO-STZ
Body weight (g)	28.95 ± 1.86	27.11 ± 2.33	32.76 ± 1.84	41.44 ± 2.40
Blood glucose (mg/dL)	183.7 ± 14.01	288.8 ± 13.80	225.5 ± 17.87	316.0 ± 27.56
Triglycerides (mg/dL)	38.13 ± 11.90	63.01 ± 24.58	262.61 ± 36.79	211.56 ± 45.74
Cholesterol (mg/dL)	45.91 ± 4.37	44.54 ± 5.13	177.21 ± 18.38	142.53 ± 32.94
LDL-cholesterol (mg/dL)	56.54 ± 19.09	53.72 ± 12.39	107.42 ± 23.63	163.97 ± 27.69
HDL-cholesterol (mg/dL)	52.65 ± 19.32	26.27 ± 8.67	142.96 ± 17.23	131.20 ± 57.78

## Data Availability

Data are available in the article or upon a reasonable request to the corresponding author.
